# Supplementary material for: Introducing spatial availability, a singly-constrained measure of competitive accessibility
Source: PLoS One. 2023 Jan 20;18(1):e0278468. doi: 10.1371/journal.pone.0278468 (PMC9858359; doi:10.1371/journal.pone.0278468)
Supplement: S1 Appendix — (PDF) [file pone.0278468.s001.pdf]

Appendix A for: Introducing spatial availability, a singly-constrained measure of competitive accessibility

Anastasia Soukhov [1] *, Antonio Páez [1], Christopher D. Higgins [2], Moataz Mohamed [3]

1 School of Earth, Environment and Society, McMaster University, Hamilton, ON, L8S 4K1, Canada
2 Department of Geography & Planning, University of Toronto Scarborough, 1265 Military Trail, Toronto, ON M1C 1A4
3 Department of Civil Engineering, McMaster University, Hamilton, ON, L8S 4K1, Canada

* Corresponding author: soukhoa@mcmaster.ca

Appendix A

In this appendix, we solve for spatial availability per capita (v_i) for population center A (Shen's synthetic example as discussed in Section 2.3) to demonstrate its mathematical equivalence to the Shen-type accessibility measure (a_i). The demonstration is shown in the following four steps.

First step: the population-based balancing factor F_i^p used in V_i is defined as:

$$F_i^p = \frac{P_i^\alpha}{\sum_i^N P_i^\alpha}$$

For population center A, F_i^p is equal to:

$$F_A^p = \frac{P_A^\alpha}{P_A^\alpha + P_B^\alpha + P_C^\alpha}$$

Second step: the impedance-based balancing factor F_{ij}^c in V_i is defined as:

$$F_{ij}^c = \frac{f(c_{ij})}{\sum_{i=A}^N f(c_{ij})}$$

In this synthetic example, combinations of workers from population center A are permitted to go to all employment centers (1, 2, 3), so their relative impedance value is experienced in all of the nine OD trip combinations. Therefore, all nine F_{ij}^c are computed as follows, since they all consider the impact of population center A trip combinations (i.e., either $A1$, $A2$, $A3$).

$$F_{A1}^c = \frac{f(c_{A1})}{f(c_{A1}) + f(c_{B1}) + f(c_{C1})}$$

$$F_{B1}^c = \frac{f(c_{B1})}{f(c_{A1}) + f(c_{B1}) + f(c_{C1})}$$

$$F_{C1}^c = \frac{f(c_{C1})}{f(c_{A1}) + f(c_{B1}) + f(c_{C1})}$$

$$F_{A2}^c = \frac{f(c_{A2})}{f(c_{A2}) + f(c_{B2}) + f(c_{C2})}$$

$$F^c_{B2} = \frac{f(c_{B2})}{f(c_{A2}) + f(c_{B2}) + f(c_{C2})}$$

$$F^c_{C2} = \frac{f(c_{C2})}{f(c_{A2}) + f(c_{B2}) + f(c_{C2})}$$

$$F^c_{A3} = \frac{f(c_{A3})}{f(c_{A3}) + f(c_{B3}) + f(c_{C3})}$$

$$F^c_{B3} = \frac{f(c_{B3})}{f(c_{A3}) + f(c_{B3}) + f(c_{C3})}$$

$$F^c_{C3} = \frac{f(c_{C3})}{f(c_{A3}) + f(c_{B3}) + f(c_{C3})}$$

Third step: when the balancing factors (F_i^p and F_{ij}^c) concerning population center A are assembled and divided by P_i, the denominators of the denominators cancel out. The following equation is the assigned general form, with the strike-through indicating which values cancel out:

$$v_i = \sum_j \frac{O_j}{P_i^\alpha} \frac{\frac{P_i^\alpha}{\cancel{\sum_i^N P_i^\alpha}} \cdot \frac{f(c_{ij})}{\cancel{\sum_i^N f(c_{ij})}}}{\sum_i^N \frac{P_i^\alpha}{\cancel{\sum_i^N P_i^\alpha}} \cdot \frac{f(c_{ij})}{\cancel{\sum_i^N f(c_{ij})}}}$$

To demonstrate that the strike-through terms cancel out, the following following terms for v_A are subbed into the general form:

$$v_A = \frac{O_1}{P_A^\alpha}\left(\frac{\frac{P_A^\alpha}{P_A^\alpha+P_B^\alpha+P_C^\alpha} \cdot \frac{f(c_{A1})}{f(c_{A1})+f(c_{B1})+f(c_{C1})}}{\frac{P_A^\alpha}{P_A^\alpha+P_B^\alpha+P_C^\alpha} \cdot \frac{f(c_{A1})}{f(c_{A1})+f(c_{B1})+f(c_{C1})} + \frac{P_A^\alpha}{P_A^\alpha+P_B^\alpha+P_C^\alpha} \cdot \frac{f(c_{B1})}{f(c_{A1})+f(c_{B1})+f(c_{C1})} + \frac{P_A^\alpha}{P_A^\alpha+P_B^\alpha+P_C^\alpha} \cdot \frac{f(c_{C1})}{f(c_{A1})+f(c_{B1})+f(c_{C1})}}\right)+$$

$$\frac{O_2}{P_A^\alpha}\left(\frac{\frac{P_A^\alpha}{P_A^\alpha+P_B^\alpha+P_C^\alpha} \cdot \frac{f(c_{A2})}{f(c_{A2})+f(c_{B2})+f(c_{C2})}}{\frac{P_A^\alpha}{P_A^\alpha+P_B^\alpha+P_C^\alpha} \cdot \frac{f(c_{A2})}{f(c_{A2})+f(c_{B2})+f(c_{C2})} + \frac{P_A^\alpha}{P_A^\alpha+P_B^\alpha+P_C^\alpha} \cdot \frac{f(c_{B2})}{f(c_{A2})+f(c_{B2})+f(c_{C2})} + \frac{P_A^\alpha}{P_A^\alpha+P_B^\alpha+P_C^\alpha} \cdot \frac{f(c_{C2})}{f(c_{A2})+f(c_{B2})+f(c_{C2})}}\right)+$$

$$\frac{O_3}{P_A^\alpha}\left(\frac{\frac{P_A^\alpha}{P_A^\alpha+P_B^\alpha+P_C^\alpha} \cdot \frac{f(c_{A3})}{f(c_{A3})+f(c_{B3})+f(c_{C3})}}{\frac{P_A^\alpha}{P_A^\alpha+P_B^\alpha+P_C^\alpha} \cdot \frac{f(c_{A3})}{f(c_{A3})+f(c_{B3})+f(c_{C3})} + \frac{P_A^\alpha}{P_A^\alpha+P_B^\alpha+P_C^\alpha} \cdot \frac{f(c_{B3})}{f(c_{A3})+f(c_{B3})+f(c_{C3})} + \frac{P_A^\alpha}{P_A^\alpha+P_B^\alpha+P_C^\alpha} \cdot \frac{f(c_{C3})}{f(c_{A3})+f(c_{B3})+f(c_{C3})}}\right)$$

v_A simplifies to the following:

$$v_A = \frac{O_1}{P_A^\alpha}\left(\frac{\frac{P_A^\alpha}{P_A^\alpha+P_B^\alpha+P_C^\alpha}\cdot\frac{f(c_{A1})}{f(c_{A1})+f(c_{B1})+f(c_{C1})}}{\frac{P_A^\alpha\cdot f(c_{A1})+P_A^\alpha\cdot f(c_{B1})+P_A^\alpha\cdot f(c_{C1})}{(P_A^\alpha+P_B^\alpha+P_C^\alpha)\cdot(f(c_{A1})+f(c_{B1})+f(c_{C1}))}}\right)+\frac{O_2}{P_A^\alpha}\left(\frac{\frac{P_A^\alpha}{P_A^\alpha+P_B^\alpha+P_C^\alpha}\cdot\frac{f(c_{A2})}{f(c_{A2})+f(c_{B2})+f(c_{C2})}}{\frac{P_A^\alpha\cdot f(c_{A2})+P_A^\alpha\cdot f(c_{B2})+P_A^\alpha\cdot f(c_{C2})}{(P_A^\alpha+P_B^\alpha+P_C^\alpha)\cdot(f(c_{A2})+f(c_{B2})+f(c_{C2}))}}\right)+\frac{O_3}{P_A^\alpha}\left(\frac{\frac{P_A^\alpha}{P_A^\alpha+P_B^\alpha+P_C^\alpha}\cdot\frac{f(c_{A3})}{f(c_{A3})+f(c_{B3})+f(c_{C3})}}{\frac{P_A^\alpha\cdot f(c_{A3})+P_A^\alpha\cdot f(c_{B3})+P_A^\alpha\cdot f(c_{C3})}{(P_A^\alpha+P_B^\alpha+P_C^\alpha)\cdot(f(c_{A3})+f(c_{B3})+f(c_{C3}))}}\right)$$

Notice,the denominator of the denominator is the same as the denominator of the numerator for each j (j=1, j=2, and j=3). Now, we remove those strike-through terms (as indicated at the beginning of this step) and re-write v_a as follows:

$$v_A = \frac{O_1}{P_A^\alpha}\left(\frac{P_A^\alpha\cdot f(c_{A1})}{P_A^\alpha\cdot f(c_{A1})+P_A^\alpha\cdot f(c_{B1})+P_A^\alpha\cdot f(c_{C1})}+\frac{O_2}{P_A^\alpha}\frac{P_A^\alpha\cdot f(c_{A2})}{P_A^\alpha\cdot f(c_{A2})+P_A^\alpha\cdot f(c_{B2})+P_A^\alpha\cdot f(c_{C2})}+\frac{O_3}{P_A^\alpha}\frac{P_A^\alpha\cdot f(c_{A3})}{P_A^\alpha\cdot f(c_{A3})+P_A^\alpha\cdot f(c_{B3})+P_A^\alpha\cdot f(c_{C3})}\right)$$

Fourth step: We can now cancel out one more term, P_A^α as follows:

$$v_A = \frac{O_1}{\cancel{P_A^\alpha}}\left(\frac{\cancel{P_A^\alpha}\cdot f(c_{A1})}{P_A^\alpha\cdot f(c_{A1})+P_A^\alpha\cdot f(c_{B1})+P_A^\alpha\cdot f(c_{C1})}+\frac{O_2}{\cancel{P_A^\alpha}}\frac{\cancel{P_A^\alpha}\cdot f(c_{A2})}{P_A^\alpha\cdot f(c_{A2})+P_A^\alpha\cdot f(c_{B2})+P_A^\alpha\cdot f(c_{C2})}+\frac{O_3}{\cancel{P_A^\alpha}}\frac{\cancel{P_A^\alpha}\cdot f(c_{A3})}{P_A^\alpha\cdot f(c_{A3})+P_A^\alpha\cdot f(c_{B3})+P_A^\alpha\cdot f(c_{C3})}\right)$$

Which can be expressed as:

$$v_A = \left(\frac{O_1\cdot f(c_{A1})}{P_A^\alpha\cdot f(c_{A1})+P_B^\alpha\cdot f(c_{B1})+P_C^\alpha\cdot f(c_{C1})}+\frac{O_2\cdot f(c_{A2})}{P_A^\alpha\cdot f(c_{A2})+P_B^\alpha\cdot f(c_{B2})+P_C^\alpha\cdot f(c_{C2})}+\frac{O_3\cdot f(c_{A3})}{P_A^\alpha\cdot f(c_{A3})+P_B^\alpha\cdot f(c_{B3})+P_C^\alpha\cdot f(c_{C3})}\right)$$

And generalized to be formally identical to the Shen-type accessibility measure with competition as follows:

$$v_i = a_i = \sum_j \frac{O_j\cdot f(c_{ij})}{\sum_i P_i\cdot f(c_{ij})}$$

\end{landscape} 4
